# Automated evaluation of diaphragm configuration based on chest CT in COPD patients

**DOI:** 10.1186/s41747-024-00491-9

**Published:** 2024-08-01

**Authors:** Jens T. Bakker, Jorine E. Hartman, Karin Klooster, David A. Lynch, Marieke C. van der Molen, Jean-Paul Charbonnier, Michail Tsiaousis, Rozemarijn Vliegenthart, Dirk-Jan Slebos

**Affiliations:** 1 University of Groningen, University Medical Center Groningen, Department of Pulmonary Diseases, Groningen, The Netherlands; 2 University of Groningen, University Medical Center Groningen, Groningen Research Institute for Asthma and COPD, Groningen, The Netherlands; 3 National Jewish Health, Department of Radiology, Denver, CO USA; 4 Thirona, Nijmegen, The Netherlands; 5 University of Groningen, University Medical Center Groningen, Department of Radiology, Groningen, The Netherlands

**Keywords:** Diaphragm, Lung pulmonary disease (chronic obstructive), Segmentation tool, Tomography (x-ray computed)

## Abstract

**Background:**

Severe chronic obstructive pulmonary disease (COPD) often results in hyperinflation and flattening of the diaphragm. An automated computed tomography (CT)-based tool for quantifying diaphragm configuration, a biomarker for COPD, was developed in-house and tested in a large cohort of COPD patients.

**Methods:**

We used the LungQ platform to extract the lung-diaphragm intersection, as direct diaphragm segmentation is challenging. The tool computed the diaphragm index (surface area/projected surface area) as a measure of diaphragm configuration on inspiratory scans in a COPDGene subcohort. Visual inspection of 250 randomly selected segmentations served as a quality check. Associations between the diaphragm index, Global Initiative for Chronic Obstructive Lung Disease (GOLD) stages, forced expiratory volume in 1 s (FEV1) % predicted, and CT-derived emphysema scores were explored using analysis of variance and Pearson correlation.

**Results:**

The tool yielded incomplete segmentation in 9.2% (2.4% major defect, 6.8% minor defect) of 250 randomly selected cases. In 8431 COPDGene subjects (4240 healthy; 4191 COPD), the diaphragm index was increasingly lower with higher GOLD stages (never-smoked 1.83 ± 0.16; GOLD-0 1.79 ± 0.18; GOLD-1 1.71 ± 0.15; GOLD-2: 1.67 ± 0.16; GOLD-3 1.58 ± 0.14; GOLD-4 1.54 ± 0.11) (*p* < 0.001). Associations were found between the diaphragm index and both FEV1% predicted (*r* = 0.44, *p* < 0.001) and emphysema score (*r* = −0.36, *p* < 0.001).

**Conclusion:**

We developed an automated tool to quantify the diaphragm configuration in chest CT. The diaphragm index was associated with COPD severity, FEV1%predicted, and emphysema score.

**Relevance statement:**

Due to the hypothesized relationship between diaphragm dysfunction and diaphragm configuration in COPD patients, automatic quantification of diaphragm configuration may prove useful in evaluating treatment efficacy in terms of lung volume reduction.

**Key Points:**

- Severe COPD changes diaphragm configuration to a flattened state, impeding function.
- An automated tool quantified diaphragm configuration on chest-CT providing a diaphragm index.
- The diaphragm index was correlated to COPD severity and may aid treatment assessment.

**Graphical Abstract:**

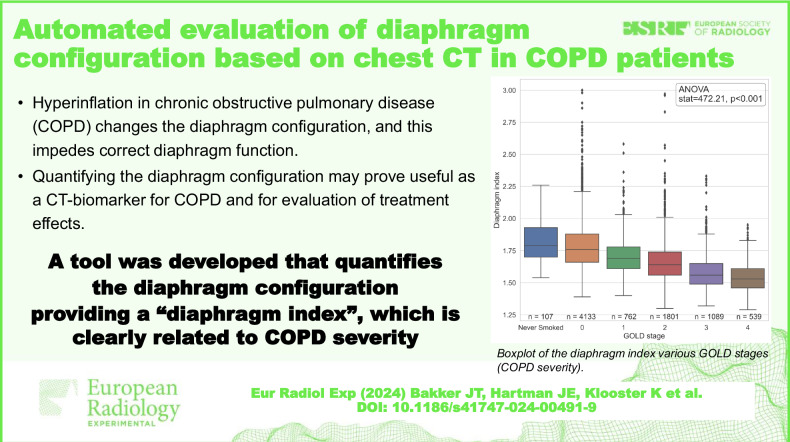

## Background

Chronic obstructive pulmonary disease (COPD) is a condition characterized by lung tissue and airway damage, resulting in reduced lung elasticity. This leads to airway and lung tissue collapse during expiration, causing trapped air and hyperinflation [2]. As a result, individuals with COPD experience symptoms like shortness of breath and decreased exercise capacity [3].

Severe hyperinflation forces the diaphragm, the primary respiratory muscle, into a mechanically disadvantaged configuration. This altered configuration is characterized by flattening and a reduced zone of apposition, where the diaphragm is directly opposed to the lower rib cage [4], and leads to pronounced symptoms of breathlessness and chest discomfort while shortening its operating length [5, 6]. Studies have shown that the generated transdiaphragmatic pressure is reduced in COPD patients compared to healthy individuals [7, 8]. Interestingly, when comparing transdiaphragmatic pressures at similar lung volumes, patients with COPD and healthy subjects demonstrated similar results [8], suggesting that the diaphragm’s unfavorable configuration plays a role in the reduced pressures.

Interventions like endobronchial valves (EBV) or lung volume reduction surgery (LVRS) aim to alleviate hyperinflation by inducing atelectasis or removing a lobe, respectively, often resulting in symptom improvement and better lung function parameters [9]. A possible factor contributing to this improvement may be a positive change in diaphragm configuration, specifically, a decrease in flattening and an increase in the zone of apposition. This appears to be supported by a study demonstrating measurable diaphragm configuration changes after LVRS [10]. This highlights the potential significance of diaphragm configuration parameters in assessing hyperinflation treatment feasibility.

Various techniques evaluating the diaphragm have been used, for example, ultrasound for assessing thickness and excursion-related parameters [11] and chest radiographs for determining diaphragm position and motion [12]. Other modalities used are fluoroscopy, magnetic resonance imaging [13, 14], and computed tomography (CT) [15]. CT is of particular interest when considering EBV or LVRS, due to its frequent use in assessing emphysema and fissure integrity [9]. Additionally, CT provides three-dimensional information on the complete diaphragm. However, extracting quantitative information poses a substantial challenge, due to the similarity in density with surrounding organs, especially the liver [16]. Therefore, many approaches involve using known landmarks to manually segment the diaphragm [17, 18].

There are automatic approaches as well, which may offer advantages in terms of speed and eliminating interobserver variability. These approaches generally involve an approximate diaphragm segmentation, by taking the lung-diaphragm intersection, which is the lowest part of the lung segmentation directly adjacent to the diaphragm [19–21]. One attempt to automatically segment the diaphragm improved upon previous attempts [19, 20] and was reported by Chang et al [21] in a small cohort, based on 16-multidetector CT, without further results reported for this method after 2016. They introduced the “diaphragm index” as a measure of diaphragm configuration. Particular details of their method remain unclear and the potential of this approach was demonstrated only by focusing on the flatness of the top part of the diaphragm. Other automatic approaches involved using additional segmentations, such as the ribcage and/or the sternum to include the zone of apposition using complex methods, for which the software is not widely available [22, 23]. Considering the difficult replication of these methods, the benefits of automation, and the potential significance of diaphragm configuration parameters within hyperinflation treatment, we aimed to develop a simplified automated method for extracting the lung-diaphragm intersection from chest CT to facilitate measurement of the diaphragm index. Additionally, we aimed to evaluate the capability of this method to differentiate COPD patients from non-smoking controls.

## Methods

### Patient population

We included a subcohort from the COPDGene study [1]. This study included self-identified non-Hispanic white and African American individuals between the ages of 45 and 80 years with a smoking history of at least 10 pack-years, except for a sample of never-smoking controls. The exclusion criteria included pregnancy, lung diseases other than COPD, and active cancer. For our analysis, we utilized the post-bronchodilator forced expiratory volume in 1 s (FEV1) and forced vital capacity (FVC) that had been collected from every participant. More details on spirometry data collection can be found in the COPDGene study design [1]. Individuals with smoking history were classified into the Global Initiative for Chronic Obstructive Lung Disease (GOLD) stages, corresponding to their FEV1/FVC and FEV1% predicted status (using Global Lung Initiative reference values) [24, 25].

For our current study, we included COPDGene participants of whom inspiratory CT-scan and FEV1 data were available. We excluded the patients that can be classified as having a “preserved ratio impaired spirometry since the status of this group of patients is unclear due to the fact that transition to normal spirometry as well as to COPD is possible [26]. Individuals with a smoking history without abnormal lung function test results were classified as GOLD-0 and included as a second control group. For our analysis, we used baseline demographics, spirometry, and CT scan data.

### CT protocol

The COPDGene cohort underwent inspiratory chest CT scanning. Scans were reconstructed with a sub-millimeter slice thickness and smooth image reconstruction kernels. More details on the CT protocol can be found in the COPDGene study design paper [1].

### Development of the diaphragm segmentation tool

To automatically calculate the diaphragm index on CT datasets, we developed a new tool. The lobe segmentation obtained through LungQ (Thirona, Nijmegen, The Netherlands) is used as input.

**Fig. 1:**
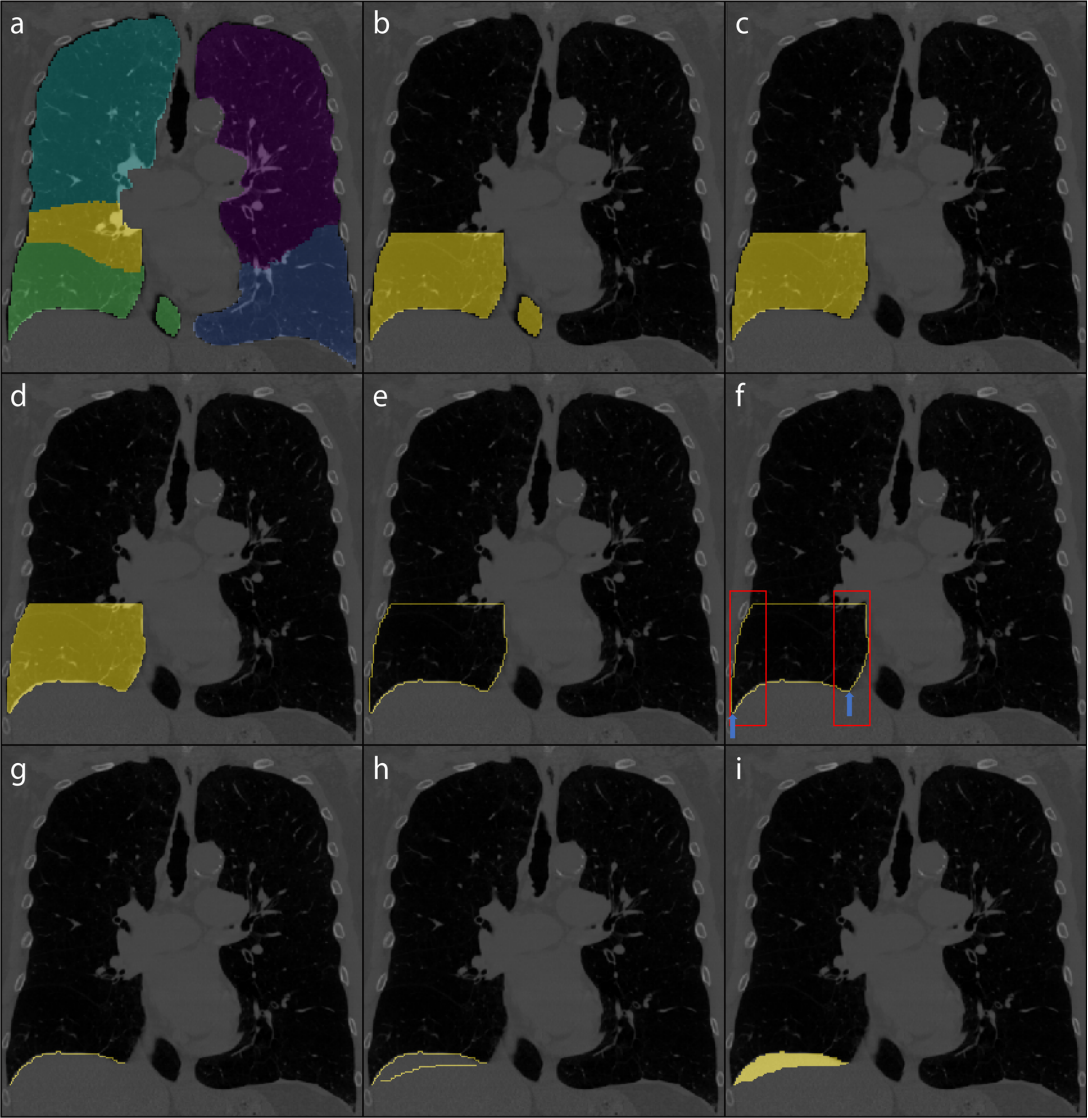
Diaphragm segmentation. For example, focusing on a single coronal slice of the right lung (each lung is processed separately and most steps are taken in the coronal slice. **a** Coronal slice of a lobe segmentation by LungQ (Thirona, Nijmegen, The Netherlands) that serves as the starting point to extract the lung-diaphragm intersection. **b** The lobe segmentation is converted into a lung segmentation and its most caudal third is retained for further processing. **c**, **d**, **e** For simplicity, the largest connected component (all the voxels within the segmentation touch each other) is kept for further processing. **f** Within the areas represented by the red boxes, the most caudal points are found, if multiple points are found at the same height the outermost is selected (signified by the blue arrows). These points are removed from the outline, separating it into two connected components. **g** The smallest component is retained, leaving the outline of the lung-diaphragm intersection. **h** The diaphragm segmentation (step **g**) from the previous slice is added to the current slice diaphragm segmentation. For visualization purposes only, instead of the previous slice, a slice from 27 slices before the current slice is chosen. **i** The ends of both diaphragm segmentations are connected and the empty space within the resulting segmentation is filled. This step ensures that the slices are connected in three dimensions, forming a fully connected object

The lobe segmentation is initially resampled to a resolution of 1 × 1 × 1 mm^3^ and separated into the left lung and right lung, which are processed independently. The process starts in a three-dimensional fashion, by selecting the lower third of each lung segmentation for further processing. Then, the subsequent steps are performed by iterating over the coronal slices of this lower third of the lung segmentation. First, every slice is checked for the amount of connected components, meaning a group of voxels that touch each other. If there are multiple connected components in a slice, the largest one is retained, and the others are discarded, in order to simplify the process. Then, an operation called “dilation” is performed [27], which adds a layer of voxels around the segmentation. The non-dilated, original segmentation can then be removed from the dilated segmentation, creating an outline of this lowest third of the segmented lung. The width of this outline is determined and used to create search areas, which are implemented on the first 25% of the width and on the last 25% of the width. Within these search areas, the most caudally present voxel is discarded from the outline. This effectively cuts the outline into two parts or two connected components. The smallest connected component is then retained as this constitutes the lung-diaphragm intersection for this particular slice. Then the iteration moves on to the next slice and repeats this process. This process is illustrated in Fig. 1.

In rare cases, the removal of the most caudal does not result in two connected components. This is mitigated by using another approach for that particular coronal slice. Particularly, the outline is traced from the most caudal midpoint outwardly in both directions. In most cases, the most caudal point on either side of the midpoint represents the outer edge of the diaphragm. However, in some cases, the diaphragm slopes in the cranial direction towards the outer edge. To verify this, the line is checked to ascend over several pixels on both sides of the most caudal point. If not, indicating this caudal point is close to the midpoint, the outermost point is chosen as the edge of the segmentation.

Some coronal slices do not contain the lung-diaphragm intersection, but are still processed in the same way as the other coronal slices, but need to be removed from the resulting segmentation. To address this issue, two filters are implemented. The first filter assesses whether the contour of the lung-diaphragm interface curved upwards towards the top (cranially) or remained flat, as anticipated. If it curved downwards towards the bottom (caudally) at the midpoint, it is removed. The second filter finds the middle of the line and its most caudal point. If the distance between these points is too short, suggesting the line was curving inwards, it is also removed.

All the remaining outlines of the lung-diaphragm intersection in the processed coronal slices are connected to the outline from the previous slice (with the exception of the first slice, for obvious reasons), for an illustrated explanation see Fig. 1g–i. If some slices are skipped due to the filters, it results in gaps in the three-dimensional segmentation of the diaphragm. The assumption is that the biggest connected area represents where the lung meets the diaphragm and any smaller areas that passed through the filter incorrectly should not be part of it. So, the segmentation is checked for multiple areas in 3D, and the largest one is considered the diaphragm segmentation.

### Diaphragm index

**Fig. 2:**
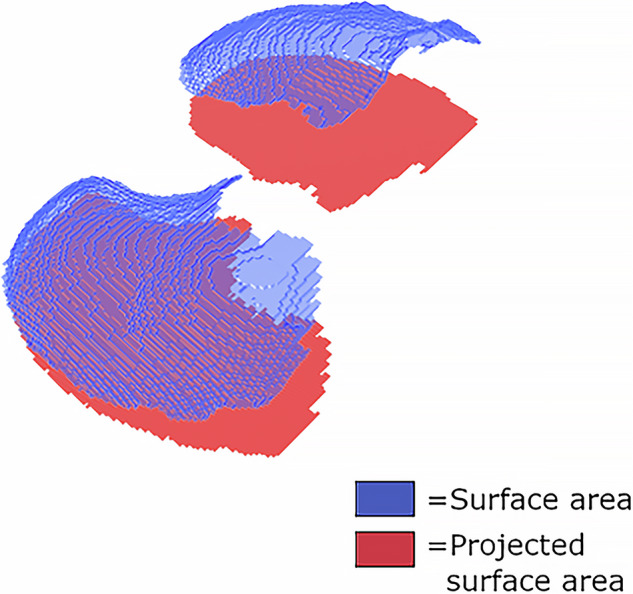
Example of a three-dimensional view of a diaphragm segmentation (in blue). The projected surface area of this diaphragm segmentation is visible in red. The diaphragm index is the ratio of the diaphragm surface area divided by the projected surface area

The diaphragm index [21] is the outcome parameter of the developed CT-based tool. It consists of the ratio between the surface area of the diaphragm and the projected surface area of the diaphragm. The projected surface area constitutes the area of the diaphragm segmentation projected in a single axial slice. Figure 2 visualizes the surface areas that make up the diaphragm index.

### Segmentation failure rate analysis

**Fig. 3:**
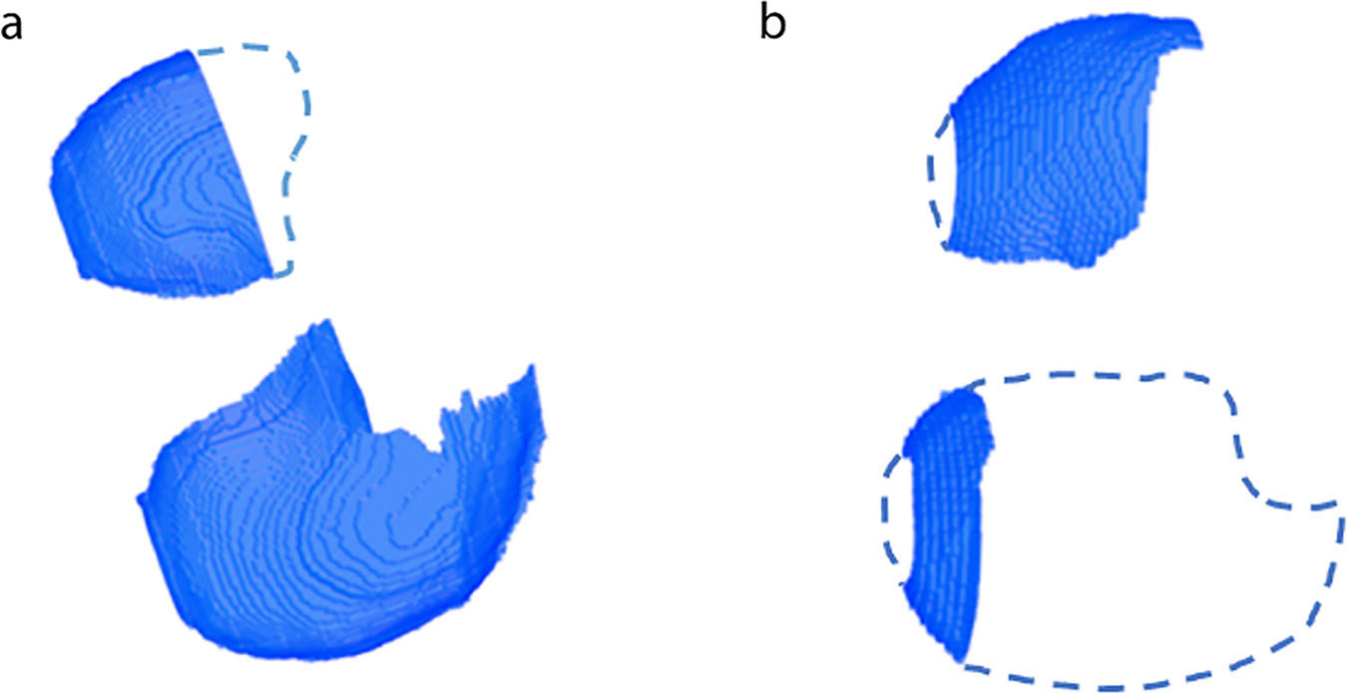
**a** Top-down view of a three-dimensional (3D) segmentation is considered a minor defect. The dashed outline roughly indicates the area that should have been included in the segmentation. **b** Top-down view of a 3D segmentation is considered a major defect. The dashed outline roughly indicates the area that should have been included in the segmentation

To inspect the failure rate of the segmentation tool, a random selection, selected by a sample function within Python, of 250 segmentations was visually inspected. Apart from that, we also checked all outliers, lying at a distance of more than 1.5 times the interquartile range beyond the upper or lower boundaries of the interquartile range in the diaphragm index associated with the respective GOLD stage [28]. The segmentation errors were divided into two categories, minor segmentation errors and major segmentation errors. The minor segmentation errors were defined by the absence of less than half of one or both hemispheres in the segmentation and/or the inclusion of a minor component that should not have been included. Major segmentation errors were defined by the absence of more than half of one or both hemispheres in the segmentation and/or the inclusion of a significant component that should have been omitted. Examples of both types of segmentation defects can be found in Fig. 3.

### Statistical analysis

The difference in diaphragm indices between GOLD stages was assessed by analysis of variance—ANOVA and a post-hoc Tukey test. Pearson correlations were calculated between the diaphragm index and FEV1% predicted and between the diaphragm index and the log transformation, in an effort to make the data suitable for linear regression, of -950 HU emphysema score (%) obtained through LungQ (Thirona, Nijmegen, The Netherlands). The correlations were also performed without the outliers. Outlier frequencies across the various GOLD stage groups were compared through a *χ*^2^ test. All statistics were performed using Python version 3.7.9 from the SciPy library version 1.7.3. A *p*-value lower than 0.05 was considered significant.

## Results

### Demographics

**Fig. 4:**
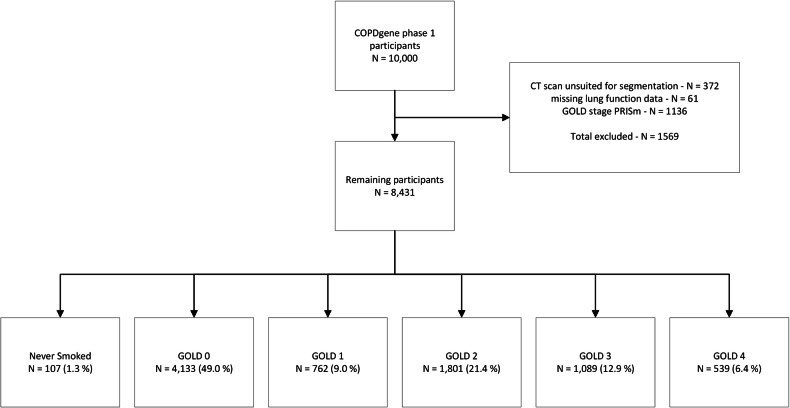
Study flowchart

The participant selection process is illustrated in Fig. 4. We included 8,431 subjects from the COPDGene cohort. The relevant demographic parameters per GOLD stage group and never-smokers are shown in Table 1.

**Table 1:** Participant characteristics

	Never smokers	GOLD 0	GOLD 1	GOLD 2	GOLD 3	GOLD 4
Number of subjects	107	4133	762	1801	1089	539
Female (%)	68.2	47.5	42.4	46.6	41.6	40.4
Age (years)	62.1 ± 9.2	56.8 ± 8.4	62.0 ± 8.9	62.5 ± 8.9	64.4 ± 8.3	64.2 ± 7.6
FEV_1_% predicted	104.0 ± 13.6	97.6 ± 11.4	91.1 ± 8.8	65.3 ± 8.5	40.4 ± 5.7	22.7 ± 4.8
Emphysema score -950 HU (%)	1.8 ± 2.6	2.0 ± 2.7	5.4 ± 5.9	7.4 ± 8.1	16.4 ± 12.5	27.1 ± 14.1

Demographics of subjects included from the COPD Gene sub-cohort. The sub-cohort is divided into groups according to the GOLD stages and never smokers. Emphysema score -950 HU, relative area below the Hounsfield unit of -950 HU

*FEV1* Forced expiratory volume in 1 s, *GOLD* Global Initiative for Chronic Obstructive Lung Disease

### Segmentation failure rate

Out of the random sample of 250 visually inspected segmentations, 23 (9.2%) were found to be faulty in some aspect, 17 (6.8%) were deemed to have a minor segmentation error, and 6 (2.4%) were deemed to have a major segmentation error. Out of 8,431 diaphragm indices measurements, 222 (2.6%) were considered outliers, 101 (45.5%) were faulty in some aspect, 80 (36.0%) had a minor segmentation defect and 21 (9.5%) had a major segmentation defect. In the GOLD-0 group, there were 99 outliers out of 4,133 participants (2.4%), while the GOLD-1 group had 21 outliers out of 762 participants (2.8%). The GOLD-2 group recorded 58 outliers out of 1801 participants (3.2%), the GOLD-3 group had 37 outliers out of 1089 participants (3.4%), and the GOLD-4 group had 7 outliers out of 539 participants (1.3%). Despite variations, the differences in outlier frequencies across the groups were not significant (*p* = 0.051, *χ*^2^ test).

### Diaphragm indices

**Fig. 5:**
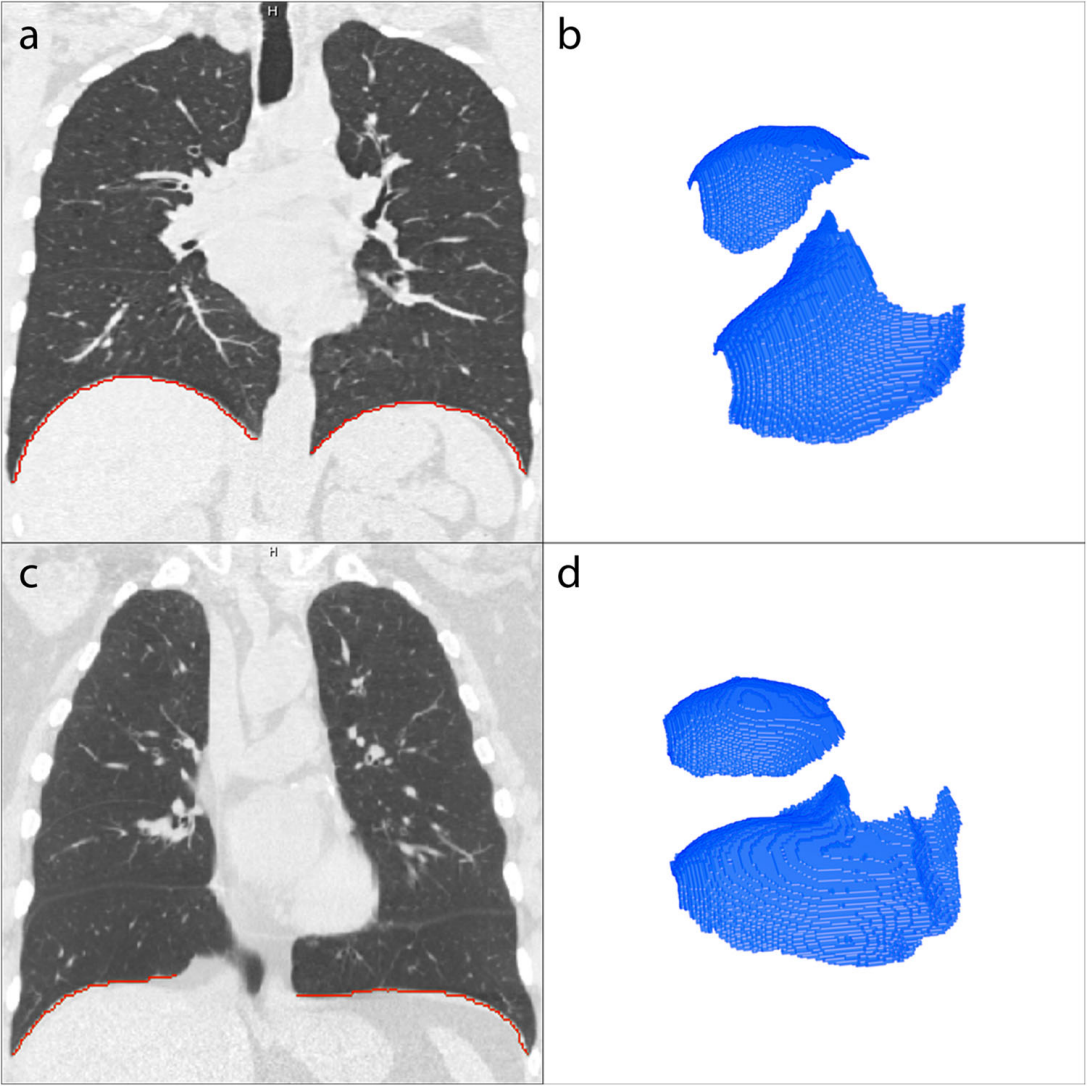
**a** Example of a coronal slice of a participant with GOLD-0. The red line represents the lung-diaphragm intersection segmentation for this particular coronal slice. **b** The entire lung-diaphragm segmentation for the same participant is depicted in **a**, with a diaphragm index of 2.25. **c** Example of a coronal slice of a participant with GOLD-4. The red line represents the lung-diaphragm intersection segmentation for this particular coronal slice. **d** The entire lung-diaphragm segmentation for the same participant is depicted in **c**, with a diaphragm index of 1.55

**Fig. 6:**
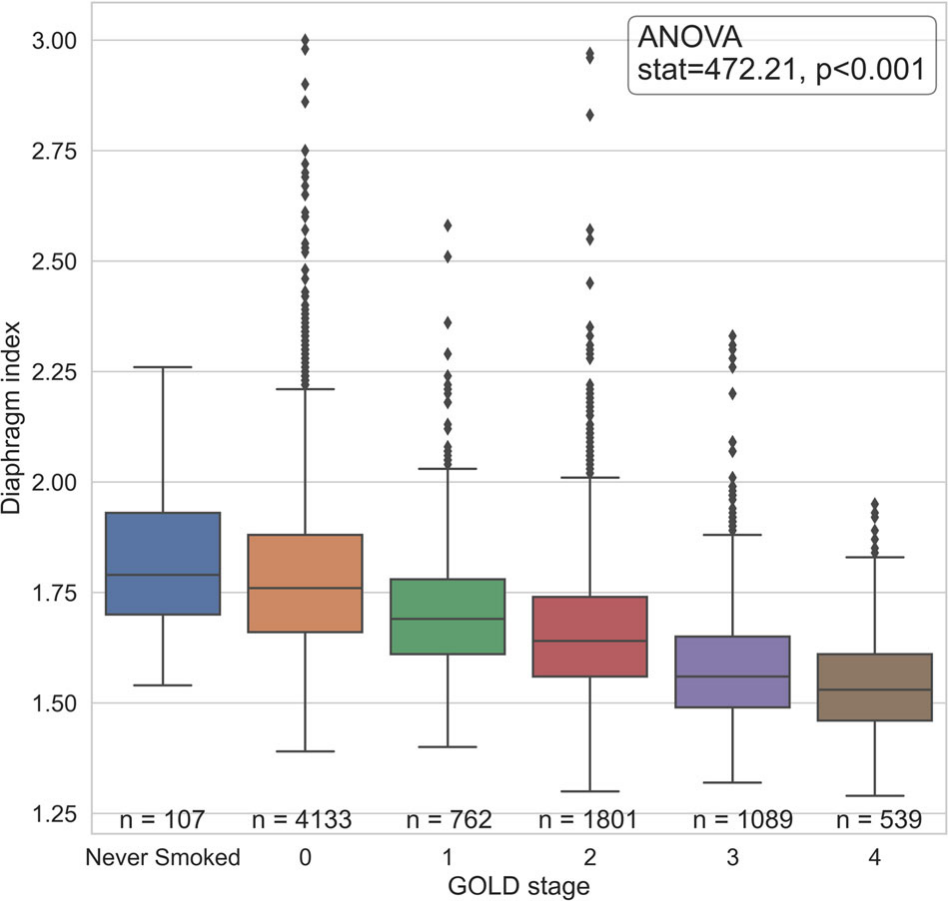
Boxplot of the diaphragm index in a group that has never smoked and groups according to the GOLD stage. The ANOVA proved significant with *p* < 0.001. The post-hoc Tukey test demonstrated a significant difference between all groups, with the exception of the Never-smoked group and the GOLD-0 group, which were not significantly different from each other

Figure 5 gives an example of two of the segmentations, with different diaphragm indices, one for GOLD-0 and one for GOLD-4. The diaphragm index was increasingly lower with higher COPD severity, ranging from 1.83 ± 0.16 (range: 1.54–2.26) for never-smokers and 1.79 ± 0.18 (1.39–3.00) for the GOLD-0 group to 1.54 ± 0.11 (1.29–1.95) for GOLD-4 group. The diaphragm index per GOLD stage is plotted in Fig. 6. There was a significant difference between all groups in diaphragm indices (*p* < 0.001), with the exception of the never-smoked group and the GOLD 0 group (Fig. 6).

**Fig. 7:**
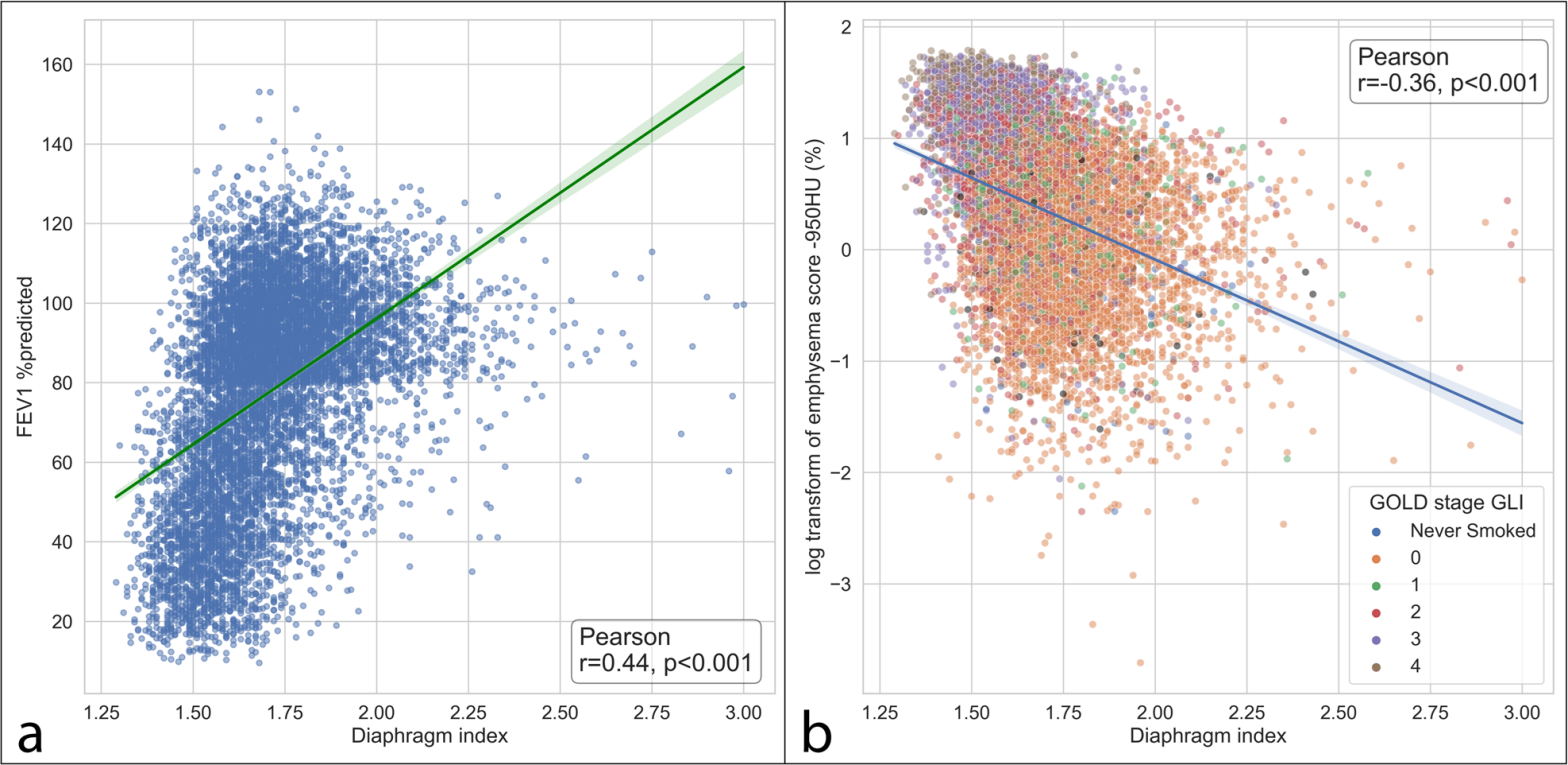
**a** Pearson’s correlation between the diaphragm index and the FEV1% predicted. **b** Pearson’s correlation between the diaphragm index and the log transformation of the -950 HU emphysema score (%)

We found a significant association between the diaphragm index and FEV1% predicted (*r* = 0.44, *p* < 0.001, Fig. 7). Furthermore, there was a significant negative association between the diaphragm index and the log-transformed CT emphysema score (*r* = -0.36, *p* < 0.001, Fig. 7). The correlation strengths increased when the outliers were filtered from the results: FEV1% predicted (*r* = 0.49, *p* < 0.001) and log-transformed emphysema score at -950 HU (*r* = -0.38, *p* < 0.001).

## Discussion

We developed a CT-based tool to evaluate the diaphragm configuration. The developed tool focused on extracting the intersection between the lung and diaphragm and measuring the corresponding diaphragm index. Using a subcohort of healthy individuals and COPD patients from the COPDGene cohort, the diaphragm index resulted to be correlated with the GOLD stage, FEV1% predicted, and emphysema score.

The study indicates a clear association between the diaphragm index and COPD severity. Specifically, there was a significant difference between the diaphragm indices for every GOLD stage. Only the never-smokers and the GOLD-0 group had not significantly different diaphragm indices. As both these groups are considered healthy, the lack of difference in diaphragm index is expected. Our findings are corroborated by the significant association between the diaphragm index and the FEV1% predicted, as well as the correlation between the diaphragm index and the log-transformed emphysema score.

Actual segmentation of the diaphragm using CT data is difficult to realize. Therefore, prior research focuses on inferring the location of the diaphragm using the surrounding anatomical structures. The main anatomical feature used is the lung. In some approaches, the zone of apposition is included, which is always derived through other anatomical structures such as the ribs. This can be done manually [17, 18] or automatically [22, 23]. Other studies used automatic techniques focusing on the lung-diaphragm intersection [19–21], adopting three-dimensional approaches rather than a per-slice approach. One involved constructing a surface mesh of the lungs to evaluate the associated normal vectors [19], allowing for the inclusion of all voxels linked to a specific direction along the lung-diaphragm intersection segmentation. Another method selected a specific point below the lung and used that point as the basis for ray projection towards the lung segmentation to extract the lung-diaphragm intersection [20]. A third approach combined both these methods using a graph cut method, using first the ray tracing to establish candidate points, subsequently examining the points for continuity of their associated normal vectors, thus refining the lung-diaphragm interface [21]. All these methods, including our own, leveraged the characteristic dome shape of the diaphragm and the sharp edge that this dome has with the rest of the lung segmentation. Our approach stands out for its simplicity compared to other methods.

Chang et al [21] reported superior segmentation quality for their method compared to the other two mentioned techniques. Unfortunately, a direct comparison with the method proposed by Chang et al is unattainable as the details about their method do not allow reproduction. For example, they did not clearly explain how the point that formed the base of the ray projection was defined. Additionally, they only correlated the diaphragm index to absolute FEV1 values in a small cohort of 30 patients with COPD and 10 healthy controls. In contrast, we studied the association of the diaphragm index with FEV1% predicted, which accounts for sex, height, and age, making it more suitable for such an association, in a larger cohort. Even so, Chang’s study and ours identified a substantial correlation between the diaphragm index and FEV1, as well as the FEV1% predicted. These results support the idea that the diaphragm index measures the configuration of the diaphragm and thereby provides information on the severity of the disease in an individual patient. Additionally, both the present study and that by Chang et al [21] demonstrated that a diaphragm segmentation that excludes the zone of apposition is still able to detect significant changes in the diaphragm of patients with hyperinflation.

The assumption is that hyperinflation causes a reduced diaphragm index, *i.e*., an impaired diaphragm. In this hypothesis, the goal of LVRS or EBV treatment would mainly be the restoration of the diaphragm configuration. This hypothesis is supported by a study showing similar trans-diaphragmatic pressures in COPD patients and healthy subjects at matched lung volumes [8]. Furthermore, lung function improves with LVRS or EBV [9], alongside enhancements in diaphragm configuration [10]. Nevertheless, there are some opposing views. It is possible that hyperinflation does lower the diaphragm index, but that this is not the cause of the lower lung function measurements. For example, it is known that the diaphragm in patients with COPD undergoes several structural tissue alterations, such as a change in muscle fiber contractility and a shift in muscle fiber type [29–31]. There are some indications that some of these alterations develop in an early stage of the disease [30, 31]. It has even been proposed that the difference in transdiaphragmatic pressures between healthy individuals and COPD patients can be explained by cellular and molecular changes alone [32]. This seems unlikely due to the improvements found in lung function by LVRS or EBV. However, other mechanisms than a change in the diaphragm configuration may still play a major role in the reduced lung functions found in patients with hyperinflation. Even if our hypothesis is correct, it is still uncertain whether the diaphragm index offers meaningful additional insights compared to regular hyperinflation measurements. Further studies are needed.

A key strength of our study is that it features a large cohort of 8,431 participants across all GOLD stages, all with sufficient size to compare diaphragm indices between groups. Furthermore, the relatively simple segmentation method resulted in an acceptable defect rate, as it likely minimally affected this analysis. This can be assumed for two reasons. First, the majority of errors can be classified as minor and are unlikely to severely affect the measured diaphragm index. Second, the correlation strengths improved when outliers, which include most errors, were removed from the data: even if segmentations with errors were filtered out entirely, the conclusions drawn would likely remain unchanged. Additionally, the diaphragm index was already verified in a smaller cohort.

Our study also has some limitations. Our segmentation method did not actually segment the diaphragm, as the similarity in the density of the diaphragm with surrounding organs, especially the liver, makes this difficult [10]. Therefore, the lung-diaphragm intersection was used. An actual segmentation of the diaphragm would be preferable, as it would not be limited to the contact area of the lungs with the diaphragm and could be used to infer information on diaphragm thickness, which has been demonstrated to be related to the pressure generation of the diaphragm in ultrasound studies [11, 33, 34]. Furthermore, the zone of apposition could not be considered, which has been claimed to be the exclusive area in which changes to the diaphragm occur in patients with hyperinflation [6]. However, we have demonstrated that the diaphragm configuration changes as well. An analysis of the frequency of defects was performed to obtain an idea of the accuracy of the method, but no defects were filtered from the data, possibly affecting the results. Repeating the analyses without outliers, which had a relatively high amount of defects, led to slightly stronger associations. The frequency of outliers was not significantly different across GOLD stages, however, the *p*-value was close to the 0.05 threshold, therefore no definitive conclusions can be made. Due to the lack of plethysmography-derived lung volume measurements in the COPDGene cohort, we were not able to associate more traditional hyperinflation measures like the percent residual volume predicted and the residual volume to total lung capacity ratio, with the diaphragm index, even though this is arguably what the diaphragm index would be most closely related to. However, hyperinflation measures are related to FEV1% predicted [35]. So, indirectly, it can be inferred that hyperinflation is related to a lower diaphragm index. However, the extent of this relationship remains unknown and would be interesting to explore further. Additionally, conclusions regarding the utility of the diaphragm index in LVRS or EBV treatment cannot be drawn as we have not yet compared diaphragm index results before and after treatment, nor have we related this measure to other outcomes. Similarly, we cannot conclude the utility of the diaphragm index in other lung diseases or conditions such as diaphragmatic paralysis, where it may also prove useful.

In the future, we plan to assess differences in the diaphragm index before and after EBV treatment in patients with severe COPD to determine if treatment induces changes in diaphragm configuration. We are interested in the relationship between hyperinflation and the diaphragm configuration as well. Additionally, we want to explore whether the diaphragm index may serve as a treatment responder criterion and possibly predict treatment responses. Furthermore, we aim to investigate how the diaphragm index can be used to define different phenotypes within COPD. Eventually, we may determine the role of factors like sex, height, age, and weight in relation to the diaphragm index. The utility of the diaphragm index in other (lung) conditions may be explored in the future as well.

In conclusion, we developed an automated CT-based tool, which is able to analyze relevant diaphragm configuration information using the diaphragm index. The study found that the diaphragm index was associated with COPD status according to GOLD classification, FEV1% predicted, and CT emphysema score.

## Data Availability

Data analyzed during the study were provided by COPDGene. Formal requests for data and analysis plans should be directed to COPDGene (www.copdgene.org). The code used to generate the results outlined in this manuscript is owned by Thirona, Nijmegen, NL. Therefore, requests for access to this code should be directed to Thirona (www.thirona.eu).

## Abbreviations

COPD: Chronic obstructive pulmonary disease
CT: Computed tomography
EBV: Endobronchial valves
FEV1: Forced expiratory volume in 1 s
FVC: Forced vital capacity
GOLD: Global Initiative for Chronic Obstructive Lung Disease
LVRS: Lung volume reduction surgery
